# Assessing the Credibility of ChatGPT on Temporomandibular Disorders

**DOI:** 10.1111/eje.70152

**Published:** 2026-03-29

**Authors:** Hilal Yilanci, Amr Anjary, Mohammed Adleh

**Affiliations:** ^1^ Department of Orthodontics Faculty of Dentistry Graduate School of Health Sciences Istanbul Medipol University Istanbul Turkey; ^2^ Division of Orthodontics, Craniofacial Biology Externship Program University of Kentucky Kentucky USA

**Keywords:** artificial intelligence, deep learning/machine learning, dental education, dental health survey(s), dental public health

## Abstract

**Introduction:**

Large Language Models such as ChatGPT are increasingly used in dental education; however, their credibility in clinical contexts remains uncertain. This study is aimed to analyse the credibility and efficacy of responses given by ChatGPT about temporomandibular disorders among dentists and dental students.

**Materials and Methods:**

Nine questions related to TMDs were posed to ChatGPT 3.5, and its responses were used to create an online survey. A total of 115 participants (60 dental students and 55 dentists) rated each response on a five‐point Likert scale. Additionally, a Delphi panel of 14 TMD specialists assessed the same responses for accuracy, completeness, and guideline adherence based on DC/TMD and AAOP criteria. Consensus was defined as ≥ 70% agreement among panellists. The Mann–Whitney *U* test was used. A significance level of *p* < 0.05 was considered.

**Results:**

The study indicates positive perceptions with median values for all questions scoring more than 4. Descriptive statistics, revealing mean scores ranging from 4.28 to 4.48. The score of each response did not show a significant difference between groups. Dental students demonstrated significantly higher total scores across all nine responses compared with the dentist group (*p* = 0.029). Delphi findings indicated strong expert consensus, with median scores ≥ 4 and interquartile ranges mostly equal to 1.

**Conclusion:**

ChatGPT 3.5 produced accurate and coherent responses about TMDs but should be used as a supplementary educational tool under professional supervision. Incorporating Delphi‐based expert validation strengthened objectivity and demonstrated the value of combining user feedback with expert consensus when assessing AI‐generated medical information.

## 1 Introduction

In recent years, the rapid advancement of Artificial Intelligence (AI), particularly in the form of Large Language Models (LLMs) like ChatGPT, has captured the interest of individuals across all age groups [1]. From young students to professionals, people are increasingly drawn to the transformative potential of AI technologies in various aspects of life [2]. However, as LLMs become more integrated into our daily routines and decision‐making processes, questions regarding their trustworthiness and credibility naturally arise [3].

At the core of the trustworthiness of LLMs like ChatGPT lies the quality of the data they learn from. LLMs rely on vast amounts of data to train their algorithms and generate responses to user queries [4]. When this data is comprehensive, diverse, and representative of the real‐world scenarios it aims to address, LLMs can produce reliable and informative outputs. However, if the data used for training is biased, incomplete, or unrepresentative, it can lead to skewed results and undermine the credibility of the LLMs [5].

The potential for enhancing patient treatment through better results and decreased errors exists with the integration of ChatGPT or other LLMs with temporomandibular disorders (TMDs) and even dental health practices in general [6]. ChatGPT can efficiently communicate with patients by understanding their problems, answering their inquiries, and giving clear instructions in an intelligible manner since it can understand and produce human‐like responses [7].

AI powered chatbots are increasingly utilized across various sectors, including customer service, education, research, and healthcare [8]. These chatbots leverage advanced natural language processing techniques to provide human‐like responses and simulate conversational interactions. ChatGPT, developed by OpenAI, represents one of the latest advancements in AI‐driven language models. Employing supervised and reinforcement learning strategies, ChatGPT has garnered significant attention within the medical community for its ability to autonomously respond to inquiries and engage in dialogue [1, 7]. Despite not being specifically designed for healthcare applications, the widespread availability of smartphones and internet access has made ChatGPT easily accessible to the general public and dentistry in particular, contributing to its growing popularity and utilization in various domains [9].

TMDs present a common yet often perplexing challenge for many patients, characterized by symptoms that can be vague and difficult to comprehend [6]. There have been previous studies that have explored ChatGPT and ScholarGPT's utility on TMD by oral surgeons. While the answers provided to simple questions related to TMD were satisfactory, the responses to more detailed technical questions were found to be inadequate [10, 11].

In addition to subjective assessments, the reliability of AI‐generated medical information can be strengthened through expert consensus methods such as the Delphi technique [12]. The Delphi method is a structured, iterative process that gathers judgements from a panel of experts to achieve consensus on complex issues where empirical evidence may be limited [13, 14]. This approach has been widely applied in medical and dental research to validate diagnostic criteria, treatment guidelines, and educational content [15, 16]. In the context of evaluating LLM‐generated responses, the Delphi process provides an objective benchmark by comparing AI outputs against expert‐validated standards. Incorporating this method ensures a more comprehensive and systematic evaluation of ChatGPT's factual accuracy, completeness, and guideline adherence in TMD‐related information.

Assessing the credibility and readability of ChatGPT on this topic by dental students could be significant, as it has not been evaluated yet, especially considering the new generation's tendency to embrace new technologies during educational programs. Accordingly, this study provides a thorough content analysis of ChatGPT's approach to disseminating details regarding TMDs, including symptoms, causes, complications, and treatments, as well as a set of inquiries pertaining to comprehension of the subject.

## 2 Materials and Methods

Ethical approval was not necessary since the study did not involve the use of human or animal materials. In this study, a series of nine questions concerning TMDs (including their causes, symptoms, treatments, complications, and prognosis) were obtained from a study evaluating ChatGPT responses about myopia, with the same purpose, and the questions were modified and adapted according to TMDs [17]. These nine questions are appropriate for evaluation or response by undergraduate dental students in their final 2 years of BDS/DDS, as they should have a basic understanding of TMDs based on their studies in oral health, anatomy, and temporomandibular joint (TMJ) function. The questions also suit general dentists (BDS/DDS) with 5 years of theoretical, practical, and clinical training. Each questioning session was fresh, avoiding any prior queries that might introduce bias into the findings, thus safeguarding the integrity of the results. The questions were posed to ChatGPT 3.5, and the obtained responses were utilized to construct a survey (Table 1). The survey design underwent a review process by the authors, comprising a single researcher with over 15 years of clinical and academic experience in orthodontics, one PhD student specializing in orthodontics, and a senior dental student.

**TABLE 1:** Questions posed to ChatGPT and responses given.

Type of question	Questions posed to ChatGPT	Responses provided by ChatGPT
General information	**Q1:** What is TMD?	**R1:** TMD refers to a group of conditions that affect the TMJ, which is the joint that connects your jaw to your skull. This disorder can cause pain and dysfunction in the jaw joint and the muscles that control jaw movement. Common symptoms of TMD include jaw pain, difficulty chewing, clicking or popping sounds when opening or closing the mouth, and stiffness in the jaw muscles. The exact cause of TMD is often unclear and may involve a combination of factors such as jaw injury, arthritis, genetics, or teeth grinding. Treatment for TMD may include lifestyle changes, medications, physical therapy, and in some cases, dental procedures
Cause	**Q2:** What causes TMD?	**R2:** The exact cause of TMD is often not clear and can vary from person to person. TMD is likely to result from a combination of factors, and potential causes include: Jaw injury: Trauma to the jaw, such as a heavy blow or whiplash, can contribute to the development of TMD. Bruxism (teeth grinding or clenching): Persistent grinding or clenching of teeth can lead to stress on the TMJ and surrounding muscles, potentially contributing to TMD. Arthritis: Various forms of arthritis, such as osteoarthritis or rheumatoid arthritis, can affect the TMJ and contribute to TMD. Genetics: There may be a genetic predisposition to developing TMD, as some individuals may be more susceptible to jaw joint problems. Poor posture: Incorrect posture, especially in the neck and upper back, can affect the jaw and contribute to TMD. Stress: Emotional stress can lead to habits like teeth clenching or grinding, which may contribute to TMD. Dental issues: Malocclusion (misalignment of the teeth or jaw), missing teeth, or other dental problems may contribute to TMD. It's important to note that for many individuals, TMD develops without a clear, identifiable cause. Additionally, certain risk factors, such as gender (as TMD is more common in women), may play a role in its development. If you suspect you have TMD, it's advisable to consult with a healthcare professional for an accurate diagnosis and appropriate management.
Symptom	**Q3:** What are the symptoms of TMD?	**R3:** The symptoms of TMD can vary among individuals, but common signs and symptoms include: Jaw pain: Discomfort or pain in the jaw joint, which may be persistent or occasional. Pain in and around the ear: TMD can cause pain in the ear, often mistaken for earaches. Facial pain: Pain or tenderness in the face, especially in the jaw area. Difficulty chewing: Some individuals with TMD may experience pain or discomfort while chewing, making it difficult to eat certain foods. Jaw clicking or popping: Audible sounds, such as clicking, popping, or grating, when opening or closing the mouth. Not everyone with TMD experiences these sounds. Limited jaw movement: Difficulty in fully opening or closing the mouth, sometimes accompanied by a feeling of the jaw getting stuck. Locking of the jaw: In some cases, the jaw may temporarily lock in an open or closed position. Muscle stiffness: Stiffness or soreness in the jaw muscles, especially upon waking up in the morning. Headaches: TMD can be associated with tension headaches, particularly in the temples. Neck and shoulder pain: Discomfort in the neck and shoulders, which may be related to muscle tension associated with TMD. It's important to note that these symptoms can overlap with other conditions, so a thorough evaluation by a healthcare professional or a specialist, such as a dentist or oral and maxillofacial surgeon, is crucial for an accurate diagnosis and appropriate management.
Prevention	**Q4:** How to prevent TMD?	**R4:** While it may not be possible to prevent TMD entirely, there are measures that individuals can take to reduce the risk of developing TMD or alleviate symptoms. Here are some general guidelines: Maintain good posture: Proper posture, especially in the neck and upper back, can help reduce strain on the jaw and decrease the risk of TMD. Avoid excessive jaw movements: Limiting activities that involve excessive jaw movements, such as chewing gum or biting on hard objects, may help prevent unnecessary stress on the temporomandibular joint. Manage stress: Since stress can contribute to teeth grinding and clenching, finding effective stress management techniques, such as relaxation exercises or counselling, may be beneficial. Practice good oral habits: Avoid excessive force while brushing your teeth, and if you grind your teeth at night, consider using a mouthguard to protect your teeth and jaw. Maintain a balanced diet: Eating a well‐balanced diet that includes foods that are easy to chew can help reduce strain on the jaw joint. Stay hydrated: Dehydration can contribute to muscle tension, so staying properly hydrated is important for overall health, including jaw health. Be mindful of jaw positioning: Avoid habits such as resting your chin on your hand or holding the telephone between your shoulder and ear, as these can contribute to strain on the jaw. Seek prompt treatment for dental issues: Addressing dental problems, such as misaligned teeth or an improper bite, early on can help prevent or mitigate TMD. It's important to note that individual factors and causes of TMD can vary, and these preventive measures may not guarantee complete prevention. If you are experiencing symptoms of TMD or are concerned about your jaw health, it's advisable to consult with a healthcare professional or a specialist, such as a dentist or oral and maxillofacial surgeon, for personalized guidance and appropriate management.
Complication	**Q5:** What are the complications of TMD?	**R5:** TMD can lead to various complications and secondary issues, including: Chronic pain: Persistent jaw pain and discomfort can significantly impact an individual's quality of life and may lead to chronic pain conditions. Headaches: TMD is often associated with tension headaches, which can be recurrent and affect daily activities. Ear problems: TMD symptoms may cause ear pain, ringing in the ears (tinnitus), or feelings of fullness or congestion. Difficulty eating: Pain and limited jaw movement can make eating difficult, potentially leading to nutritional deficiencies and weight loss. Sleep disturbances: Teeth grinding (bruxism), which is associated with TMD, can disrupt sleep and lead to sleep‐related disorders. Impact on mental health: Chronic pain and discomfort associated with TMD can contribute to anxiety, depression, and other mental health issues. Reduced jaw function: TMD may result in limitations in jaw movement, affecting speech, facial expressions, and overall jaw function. Impact on oral health: Bruxism associated with TMD can lead to dental problems, including tooth wear, fractures, and damage to dental restorations. Muscle spasms: The muscles used for chewing and jaw movement may experience spasms, leading to further pain and discomfort. Social and lifestyle impact: TMD can affect social interactions and lifestyle choices, as individuals may avoid certain activities or foods due to pain and discomfort. It's important to note that complications and the severity of symptoms can vary widely among individuals with TMD. If you suspect you have TMD or are experiencing related symptoms, seeking prompt evaluation and treatment from a healthcare professional or a specialist, such as a dentist or oral and maxillofacial surgeon, is essential to manage symptoms and prevent potential complications.
Treatment	**Q6:** Can TMD be treated?	**R6:** Yes, TMD can be treated. The approach to treatment depends on the specific cause and symptoms of the individual's condition. Treatment options for TMD may include: Conservative measures: Self‐care: Practices such as applying ice packs, eating soft foods, and avoiding extreme jaw movements. Over‐the‐counter pain medications: Non‐prescription pain relievers, such as ibuprofen or acetaminophen, may help alleviate pain and inflammation. 2 Dental treatments: Oral splints or mouthguards: These devices, often custom‐made by a dentist, can help reduce clenching or grinding of teeth and alleviate pressure on the jaw joint. Orthodontic treatment: Correcting bite issues or misalignment of teeth may be recommended to alleviate TMD symptoms. 3 Physical therapy: Exercises and stretches prescribed by a physical therapist can help improve jaw mobility and reduce muscle tension. 4 Stress management: Techniques to manage stress, such as relaxation exercises, counselling, or biofeedback, may be beneficial, especially if stress contributes to TMD symptoms. 5 Medications: Prescription medications, such as muscle relaxants or anti‐inflammatory drugs, may be recommended to alleviate pain and muscle tension. 6 Injections: In some cases, injections of corticosteroids into the jaw joint may be considered to reduce inflammation and pain. 7 Surgery: Surgical intervention is typically considered when conservative measures have not been successful, and the condition is severe. However, surgery is generally reserved for specific cases and is not the first‐line treatment. It's crucial for individuals experiencing TMD symptoms to consult with a healthcare professional or a specialist, such as a dentist or oral and maxillofacial surgeon, for an accurate diagnosis and personalized treatment plan. The effectiveness of treatment can vary from person to person, and the goal is often to manage symptoms and improve overall jaw function and quality of life.
Treatment	**Q7:** Who can treat TMD?	**R7:** TMD can be treated by various healthcare professionals, depending on the specific symptoms and causes. Here are some of the professionals who may be involved in the diagnosis and treatment of TMD: Dentists: General dentists are often the first point of contact for individuals with TMD symptoms. They can assess the condition, provide initial recommendations, and may create oral appliances like mouthguards. Prosthodontists specialize in the restoration and replacement of teeth. They may be involved in managing TMD cases related to dental prosthetics. Orthodontists focus on the alignment of teeth and jaws and may be consulted for cases where malocclusion contributes to TMD. 2 Oral and Maxillofacial Surgeons: These specialists are trained in the surgical treatment of conditions affecting the mouth, jaw, and face. In severe cases of TMD or when surgery is deemed necessary, oral and maxillofacial surgeons may be involved in the treatment. 3 Physical Therapists: Physical therapists with expertise in orofacial pain and TMD can provide exercises, stretches, and manual therapies to improve jaw function and reduce muscle tension. 4 Otolaryngologists (ENT specialists): Ear, Nose, and Throat specialists may be involved if TMD symptoms are associated with ear‐related issues. 5 Rheumatologists: In cases where arthritis is a contributing factor to TMD, rheumatologists may be consulted for diagnosis and management. 6 Primary Care Physicians: General practitioners can assess TMD symptoms, provide initial recommendations, and refer individuals to specialists as needed. It's important for individuals with TMD symptoms to seek professional evaluation and diagnosis. The choice of healthcare professional may depend on the specific symptoms, underlying causes, and the severity of the condition. A multidisciplinary approach involving collaboration among different specialists may be recommended in some cases for comprehensive care.
Natural history	**Q8:** What happens if TMD is left untreated?	**R8:** If TMD is left untreated, it can lead to various complications and potentially worsen over time. Some of the potential consequences of untreated TMD include: Chronic pain: Persistent jaw pain and discomfort can become chronic, impacting an individual's daily life and quality of life. Increased severity of symptoms: Untreated TMD symptoms, such as jaw pain, clicking or popping sounds, and limited jaw movement, may worsen over time. Headaches: TMD is often associated with tension headaches, and leaving the condition untreated may contribute to the persistence or intensification of headaches. Dental problems: Bruxism (teeth grinding) associated with TMD can lead to dental issues, including tooth wear, fractures, and damage to dental restorations. Impact on oral health: Untreated TMD may affect overall oral health, including the health of the gums and teeth. Sleep disturbances: Bruxism, a common symptom of TMD, can lead to sleep disturbances and may contribute to sleep‐related disorders. Emotional impact: Chronic pain and discomfort associated with untreated TMD can contribute to emotional issues such as anxiety and depression. Reduced jaw function: Over time, untreated TMD may lead to increased limitations in jaw movement, affecting speech, facial expressions, and overall jaw function. It's important to note that the consequences of untreated TMD can vary from person to person, and the severity of symptoms depends on factors such as the underlying causes of TMD, individual health, and lifestyle. Seeking timely evaluation and appropriate treatment from healthcare professionals or specialists is crucial to manage TMD symptoms effectively and prevent potential complications.
Prognosis	**Q9:** What is the prognosis for TMD?	**R9:** The prognosis for TMD varies widely among individuals and depends on factors such as the underlying causes, the severity of symptoms, and the effectiveness of the chosen treatment. In many cases, TMD is a manageable condition, and with appropriate care, individuals can experience relief from symptoms. However, it's important to note that TMD is a complex and multifactorial condition, and the prognosis can be influenced by various factors: Early intervention: Timely diagnosis and treatment initiation can contribute to a more favourable prognosis. Conservative measures, such as self‐care practices, lifestyle modifications, and non‐invasive treatments, are often effective in managing mild to moderate cases. Individual response to treatment: Responses to different treatments can vary widely among individuals. Some may find relief with simple self‐care measures, while others may require more comprehensive interventions, including dental treatments, physical therapy, or medications. Underlying causes: Identifying and addressing the underlying causes of TMD, such as bruxism, misalignment of the jaw, or arthritis, can impact the prognosis. Targeting these specific factors can lead to better outcomes. Compliance with treatment: Adherence to recommended treatments and lifestyle modifications can influence the success of TMD management. This includes using prescribed oral appliances, performing exercises, and following any other therapeutic recommendations. Multidisciplinary approach: In some cases, a multidisciplinary approach involving collaboration between various healthcare professionals, such as dentists, physical therapists, and oral and maxillofacial surgeons, may be necessary for comprehensive care. It's important for individuals with TMD to work closely with their healthcare professionals to develop a personalized treatment plan and to communicate any changes in symptoms or concerns. While TMD can be a chronic condition, many people find effective ways to manage their symptoms and improve their overall jaw function and quality of life.

Abbreviations: TMD, temporomandibular disorder; TMJ, temporomandibular joint.

Moreover, to maintain confidentiality and ethical standards, all collected data were anonymized and handled following privacy regulations. The responses provided by ChatGPT were evaluated through a Google Form questionnaire, with a total of 115 participants, comprising 55 dentists and 60 dental students. Each participant independently rated the quality of each response on a Likert scale, where higher scores indicated greater quality of information, with ratings ranging between (1 = very poor; 2 = poor; 3 = acceptable; 4 = good; 5 = very good). The scores of each response were summed to obtain the total score.

### 2.1 Delphi Panel Procedure

To complement the subjective Likert evaluations provided by students and general dentists, an expert‐based validation was incorporated through a Delphi panel. The panel consisted of 14 specialists in TMD and orofacial pain, each with at least 5 years of clinical and/or academic experience. The experts independently assessed ChatGPT's responses across three dimensions: accuracy (scientific and clinical correctness), completeness (coverage of essential aspects), and guideline adherence (consistency with Diagnostic Criteria for Temporomandibular Disorders‐DC/TMD and the American Academy of Orofacial Pain‐AAOP guidelines).

The Delphi process consisted of two iterative rounds. In the first round, all experts independently rated each response. In the second round, they were presented with a statistical summary (mean and range of group scores) and were invited to reconsider their ratings based on the aggregated feedback. Consensus was defined as ≥ 70% agreement among panellists on a given score. All evaluations were anonymous, and only aggregated data were analyzed. The consensus ratings from the Delphi panel served as expert‐validated benchmarks, providing an objective comparison against the subjective assessments of dental students and general dentists.

### 2.2 Statistical Analysis

The data were analysed using IBM SPSS (version 23; IBM, Armonk, NY). Normal distribution of the data was assessed using Kolmogorov–Smirnov and Shapiro–Wilk tests. For non‐normally distributed data comparisons between paired groups, the Mann–Whitney *U* test was used. Analysis results were presented as mean ± standard deviation and median (minimum—maximum). A significance level of *p* < 0.05 was considered.

## 3 Results

Table 1 shows the 9 questions about TMDs posed to ChatGPT along with the generated responses. Table 2 presents descriptive statistics for the participants' gender distribution and their scores to responses labelled R1 through R9, which collectively represent the total of 9 responses, along with the total score. The mean scores for each response ranged from 4.28 to 4.48, with scores spanning from 1 to 5. Overall, the mean total score was 39.59 ± 5.04, and a median score of 40, reflecting a range from 25 to 45.

**TABLE 2:** Descriptive statistics.

Gender	Frequency	Percentage
Female	65	56.5%
Male	50	43.5%

	Mean ± SD	95% CI	Median (min–max)
R1	4.28 ± 0.77	4.14: 4.42	4 (2–5)
R2	4.37 ± 0.79	4.23: 4.52	5 (1–5)
R3	4.48 ± 0.69	4.35: 4.61	5 (2–5)
R4	4.47 ± 0.7	4.34: 4.6	5 (2–5)
R5	4.43 ± 0.75	4.3: 4.57	5 (2–5)
R6	4.37 ± 0.73	4.24: 4.51	5 (2–5)
R7	4.43 ± 0.82	4.28: 4.58	5 (2–5)
R8	4.43 ± 0.75	4.29: 4.56	5 (2–5)
R9	4.33 ± 0.85	4.17: 4.49	5 (2–5)
Total score	39.59 ± 5.04	38.66: 40.52	40 (25–45)

Abbreviations: Min—Max, minimum—maximum; SD, standard deviation.

The score distribution presented in Figure 1 indicates generally positive evaluations for ChatGPT's responses, with most ratings clustered toward the higher end of the scale. This trend suggests overall satisfaction among participants but also highlights some variation that reflects differences in perceptions between dentists and dental students. The survey responses, rated on a scale of 1–5, indicate generally positive perceptions (Figure 1).

**FIGURE 1:**
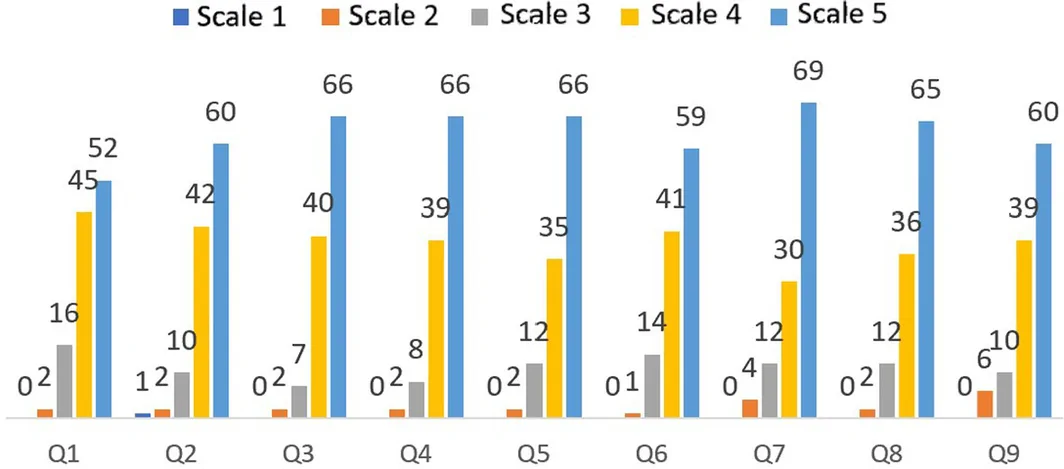
Score distribution among questions.

Each response's score was compared between dental students and dentists (Table 3). Overall, the results indicate that there are some differences in the scores between the groups, but not statistically significant. Total mean score was significantly higher for dental students (40.25 ± 5.41) compared to dentists (38.87 ± 4.53) (Figure 2).

**TABLE 3:** Comparison of scores between the groups.

	Dental student (*n* = 60)			Dentist (*n* = 55)			*p*
	Mean ± SD	95% CI	Median (min—max)	Mean ± SD	95% CI	Median (min—max)	
R1	4.37 ± 0.78	4.17: 4.57	5 (2–5)	4.18 ± 0.75	3.98: 4.38	4 (2–5)	0.130
R2	4.4 ± 0.85	4.18: 4.62	5 (1–5)	4.35 ± 0.73	4.15: 4.54	4 (2–5)	0.424
R3	4.53 ± 0.72	4.35: 4.72	5 (2–5)	4.42 ± 0.66	4.24: 4.6	4 (2–5)	0.177
R4	4.6 ± 0.59	4.45: 4.75	5 (3–5)	4.33 ± 0.79	4.11: 4.54	4 (2–5)	0.058
R5	4.45 ± 0.79	4.25: 4.65	5 (2–5)	4.42 ± 0.71	4.23: 4.61	5 (2–5)	0.569
R6	4.48 ± 0.72	4.3: 4.67	5 (3–5)	4.25 ± 0.73	4.06: 4.45	4 (2–5)	0.055
R7	4.55 ± 0.77	4.35: 4.75	5 (2–5)	4.29 ± 0.85	4.06: 4.52	5 (2–5)	0.057
R8	4.52 ± 0.77	4.32: 4.72	5 (2–5)	4.33 ± 0.72	4.13: 4.52	4 (3–5)	0.076
R9	4.35 ± 0.86	4.13: 4.57	5 (2–5)	4.31 ± 0.84	4.08: 4.54	4 (2–5)	0.669
Total score	40.25 ± 5.41	38.85: 41.65	41.5 (25–45)	38.87 ± 4.53	37.65: 40.1	39 (26–45)	0.029^a^

Abbreviations: Min—Max, minimum—maximum; SD, standard deviation.

^a^ Mann–Whitney *U* test, *p* < 0.05.

**FIGURE 2:**
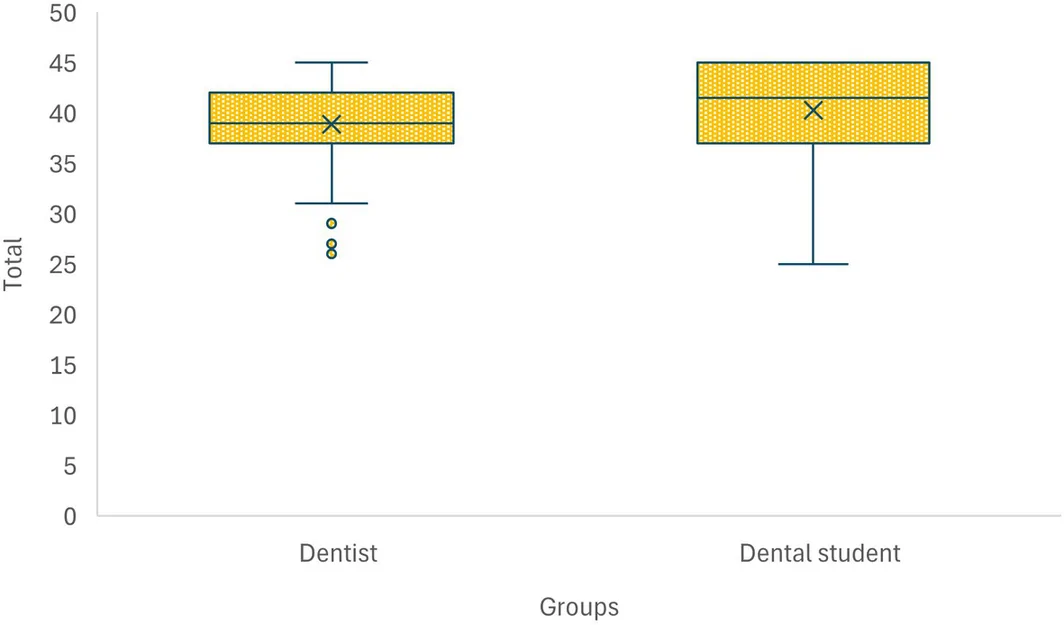
Box plot for the total scores of groups.

Analysis of the Delphi panel results demonstrated a high level of agreement among experts across all three dimensions. For Accuracy, median scores ranged from 4 to 5 with IQR values mostly equal to 1, indicating strong consensus. The highest mean score was observed for Q3 (4.64 ± 0.50), while the lowest was for Q9 (4.07 ± 1.00), which showed comparatively lower agreement (IQR = 2). In the Completeness dimension, mean scores ranged from 3.79 to 4.50, again reflecting generally favorable evaluations with strong agreement (median = 4–5). Similarly, Guideline adherence scores varied between 3.71 and 4.43, with most items showing tight interquartile ranges (IQR = 1). Overall, the consistently high median and mean values across dimensions indicate that experts rated ChatGPT's responses as accurate, comprehensive, and largely consistent with clinical guidelines.

## 4 Discussion

Educating patients about available treatments is essential for efficient care delivery, yet access to professionals for discussion can be limited [15, 18]. AI‐powered chatbots built on large language models (LLMs) offer a practical way to bridge this gap by supporting patient education and providing concise, explainable information to learners and clinicians, including on TMD topics [19, 20]. Prior evaluations show that LLM performance can be high for health information tasks, but also variable across platforms (e.g., ChatGPT, Gemini/Bard, Bing), which is relevant for dental education and evidence‐based use [20, 21, 22]. We therefore focused on ChatGPT because of its widespread availability in dentistry education research and the free access of version 3.5, enabling broad, real‐world use by students and dentists [23]. At the same time, credibility concerns remain—LLMs may produce inaccurate or misleading content—so outputs should be appraised and contextualized within professional standards, particularly in healthcare settings [1, 24].

In this study, participants evaluated ChatGPT's responses to nine key questions about TMDs using the Likert scale. The overall mean score (39.59 ± 5.04) indicated that the general impressions were good. The mean item scores ranged from 4.28 to 4.48. The results indicate that both student and dentist participants rated ChatGPT's explanations as clear, relevant, and adequately aligned with established principles of TMD. The small deviation in the two groups, with students rating slightly higher, reflects that ChatGPT provides consistent basic information that is applicable for both academic and early clinical use.

This may suggest that students, being more familiar with digital technologies and open to AI‐assisted learning tools, rate ChatGPT more favourably than dentists. Dentists, based on their clinical experience, may assess responses with higher standards for nuance or clinical specificity. Of interest, several items approached statistical significance, reflecting subtle trends in how different professional groups perceive AI‐generated content. These subtle trends may indicate specific areas where ChatGPT meets or does not meet clinicians' expectations and may guide refinements in AI‐assisted dental education [25].

The dentist group's median scores fluctuated between 4 “good” and 5 “very good”. This consistency suggests that the AI model could be proficient at providing informative and coherent responses regarding TMDs. However, it's worth noting that while the responses were of high quality, the questions posed were somewhat broad in scope. Our questions were intentionally designed to be straightforward and focused on fundamental knowledge of the TMD, covering general information, prognosis, aetiology, symptoms, prevention, complications, and natural history. This approach ensures that the questions assess essential and widely recognized concepts related to TMDs, promoting a foundational understanding that is crucial for both academic and clinical competence [26].

Balel's study in 2023 has reported similar results, wherein ChatGPT tends to show good performance in patient‐oriented questions related to oral and maxillofacial surgery but is not reliable for the complex technical questions [10]. This further elucidates that though ChatGPT can convey basic concepts well and enhance patient understanding, its limitation in deeper clinical reasoning raises the need for AI models specifically trained on academic datasets of dentistry and medicine [10].

While LLMs are able to provide an explanation that can be coherent and accessible, several limitations must be realized in the use of LLMs for TMD‐related education and patient communication. The accuracy, incompleteness, or misleading nature of the content should be considered, particularly for multisystemic conditions such as TMDs, which have symptom overlap with otologic and musculoskeletal disorders and thus are at increased risk of misinterpretation [24, 27, 28, 29]. LLMs cannot diagnose, provide individualized clinical judgement, or use contextual reasoning and thus cannot substitute for professional examination or treatment planning [1, 20]. Performance varies across platforms and model versions, and rapid model updates may introduce new inconsistencies. Thus, users must confirm information that is deemed uncertain and interpret AI‐generated responses with caution, particularly when misinformation may have disastrous consequences in healthcare settings [10, 11, 30].

ChatGPT provides an advanced learning platform that supports students' educational development by adapting and personalizing content to meet individual needs. It appears to be a suitable supplementary learning tool when used under appropriate supervision, although users should remain aware of its limitations.

While the study included two relevant groups—senior dental students and general dentists—this sample may not fully represent the broader dental community. Future research should therefore aim for more diverse and randomized populations to enhance the generalizability of findings. Because demographic variables such as gender and years of clinical experience were unevenly distributed, especially among the students, subgroup or regression analyses were not feasible. Similar imbalances have been noted in studies examining dental students' attitudes toward artificial intelligence [31]. Larger and more demographically balanced samples will be necessary to identify predictors of ChatGPT perception across different segments of the dental community.

Furthermore, we encountered practical challenges related to the length and complexity of the survey material. ChatGPT's long, detailed responses substantially increased reading time for both dentists and dental students, a difficulty that has also been reported in previous studies evaluating AI‐generated content in dentistry [10, 11]. Excessively long AI‐generated text may contribute to participant fatigue and reduce the precision of assessments; future studies would benefit from more concise, targeted question formats or summary outputs to limit cognitive overload, consistent with broader evidence on cognitive load in preclinical dental training [32].

## 5 Conclusion

Looking ahead, future research in AI‐based monitoring for TMDs using ChatGPT should prioritize larger‐scale studies with diverse participant populations to enhance generalizability. Including TMDs specialists in our study could have achieved a more precise evaluation. However, this opens the door for further investigation to be conducted in the future, allowing for a deeper and more specialized analysis of the subject matter. Longitudinal data collection and prospective study designs can provide valuable insights into evolving attitudes toward AI technology in TMDs monitoring. Additionally, refining AI algorithms for improved diagnostic accuracy and exploring innovative data integration methods can maximize clinical utility in dental practice. Longitudinal studies tracking patient outcomes post‐implementation can offer valuable insights into real‐world impact. Embracing these directions can enhance TMDs diagnosis, treatment, and patient care delivery.

The development of dentistry in particular and public health in general has greatly benefited from AI. In order to evaluate ChatGPT's trustworthiness in disseminating information on TMDs, including symptoms, causes, complications, and treatments, this study examines its discourse on the subject. It is acknowledged that the duration of the survey affects score reliability and highlights areas for improvement as well as the need for additional research. The study uses content analysis to look into ChatGPT's presentation of TMDs and contains comprehension questions to gauge participant understanding. Because of its limitations, ChatGPT should be used as a supplementary tool rather than as a main source of information. It can be concluded that ChatGPT concerning TMDs could be applied under the supervision of specialists in the field, which can help patients have a better understanding of TMDs.

## Author Contributions

Conceptualization: H.Y., A.A., M.A. Data curation: H.Y. Formal analysis: H.Y. Funding acquisition: M.A., A.A. Investigation: H.Y., A.A., M.A. Methodology: H.Y., A.A. Project administration: A.A., M.A. Software: A.A. Resources: H.Y. Supervision: H.Y. Validation: H.Y., A.A., M.A. Visualization: H.Y., M.A., A.A. Writing – original draft: H.Y. Writing – review and editing: H.Y., A.A., M.A.

## Funding

The authors have nothing to report.

## Consent

The authors have nothing to report.

## Conflicts of Interest

The authors declare no conflicts of interest.

## Data Availability

The data that support the findings of this study are available from the corresponding author upon reasonable request.
